# Differences between Spectro-Temporal Receptive Fields Derived from Artificial and Natural Stimuli in the Auditory Cortex

**DOI:** 10.1371/journal.pone.0050539

**Published:** 2012-11-27

**Authors:** Jonathan Laudanski, Jean-Marc Edeline, Chloé Huetz

**Affiliations:** 1 Centre de Neurosciences Paris-Sud (CNPS), CNRS UMR 8195, Orsay, France; 2 Centre de Neurosciences Paris-Sud, Université Paris-Sud, Orsay, France; University of Salamanca- Institute for Neuroscience of Castille and Leon and Medical School, Spain

## Abstract

Spectro-temporal properties of auditory cortex neurons have been extensively studied with artificial sounds but it is still unclear whether they help in understanding neuronal responses to communication sounds. Here, we directly compared spectro-temporal receptive fields (STRFs) obtained from the same neurons using both artificial stimuli (dynamic moving ripples, DMRs) and natural stimuli (conspecific vocalizations) that were matched in terms of spectral content, average power and modulation spectrum. On a population of auditory cortex neurons exhibiting reliable tuning curves when tested with pure tones, significant STRFs were obtained for 62% of the cells with vocalizations and 68% with DMR. However, for many cells with significant vocalization-derived STRFs (STRF_voc_) and DMR-derived STRFs (STRF_dmr_), the BF, latency, bandwidth and global STRFs shape differed more than what would be predicted by spiking responses simulated by a linear model based on a non-homogenous Poisson process. Moreover STRF_voc_ predicted neural responses to vocalizations more accurately than STRF_dmr_ predicted neural response to DMRs, despite similar spike-timing reliability for both sets of stimuli. Cortical bursts, which potentially introduce nonlinearities in evoked responses, did not explain the differences between STRF_voc_ and STRF_dmr_. Altogether, these results suggest that the nonlinearity of auditory cortical responses makes it difficult to predict responses to communication sounds from STRFs computed from artificial stimuli.

## Introduction

A major goal in auditory neuroscience is to characterize how communication sounds are represented in the auditory pathway, particularly at the cortical level. Speech, birdsongs and vocalizations are, spectrally and temporally, highly structured. A minimal prerequisite to unravel the neural representation of these sounds is to determine which spectro-temporal components drive auditory neurons’ responses.

The spectro-temporal receptive field (STRF) is probably the most commonly used model to describe the way complex stimuli are processed by auditory cortex neurons. Originally, STRFs were estimated using a reverse correlation of the neuron’s response to white noise [1]–[3]. The model is linear and relies on the stimuli statistics up to the second order [4], [5]. Due to the poor response elicited by white noise in the auditory cortex, families of synthetic stimuli have been preferred to characterize STRFs. These synthetic stimuli are commonly based on ripples (i.e. a sound modulated sinusoidally in the temporal and spectral domains: see [5]–[11]) or on random trains of pure tones [12]–[16]. Although more spectro-temporally complex than pure tones, these synthetic stimuli are still very different from conspecific vocalizations, both form the acoustical and the behavioral perspectives.

By definition, the STRF is a linear approximation of the neural response and theoretical drawbacks exist when computing STRFs of a nonlinear neural response using sounds with high order statistics [17]. These challenging nonlinearities of the responses have been sometimes tackled by an artificial stimulus design [5], or by the means of new analysis techniques [18], [19]. However, it is still not clear how STRF derived from artificial and natural sounds differ, and which nonlinearities might explain these differences.

Indeed, only a few studies have tested how linear models of auditory processing, computed using artificial stimuli, generalize to natural stimuli. In the avian auditory system, several studies have shown that STRFs can be used to describe how neurons extract auditory information from conspecific songs [4], [20], [21]. So far, studies performed in the mammalian auditory cortex have used either sets of natural stimuli [22], [23] or speech sounds to quantify STRFs of auditory cortex neurons [16], [24].

The present study compares the STRFs of guinea pig auditory cortex neurons computed from conspecific vocalizations and from dynamic broadband noises. The importance of vocal communication in guinea pig has been pointed out in pioneering studies [25]–[27] and the rich repertoire of the guinea pig when living in large colonies makes this animal a particularly well suited model for studying the neural representation of communication sounds. Here, the differences between STRF calculated from vocalizations vs. from DMRs were assessed by a similarity index, by their predictive power and by classical parameters such as best frequencies, latencies and bandwidths. To go further, we also studied the influence of our sets of stimuli using a linear spiking model and analyzed the impact of bursts, which constitute part of the nonlinearity of cortical neurons’ responses.

## Methods

### Animal Preparation and Recording Procedures

Experiments were performed on 10 adult pigmented guinea-pigs (390–650 g; national authorization N° 91–271 to conduct animal research, specifically approved by the CNRS and Paris-Sud University) anesthetized by an initial injection of urethane (1.2 g/kg, i.p.) preceded by a dose of Diazepam (6 mg/kg, i.p.). Additional doses (0.5 g/kg, i.p.) of urethane were systematically delivered when reflex movements were observed after pinching the hindpaw (usually twice during a given recording session). The body temperature was maintained around 37 C° by a heating pad throughout all the experiment. The trachea was cannulated and a local anesthetic (Xylocaine, 2%) was infiltrated in each wound. The stereotaxic frame supporting the animal was placed in a sound attenuating chamber (IAC, model AC2).

A large opening was made in the temporal bone and very small slits (200 µm) were made in the dura matter under microscopic control. A diagram of the vasculature pattern was drawn and the primary field (AI) location was first estimated based on those observed in our previous studies [28]–[30]. A mapping of the cortical surface was made to confirm the location of AI: neuronal clusters were recorded with low impedance (<1 MΩ) electrodes until a progression from low to high frequency was observed in the caudo-rostral direction [31]. At a particular cortical site, the first electrode penetration was made with a tungsten microelectrode (>8 MΩ) and the following ones (made at close vicinity but probably corresponding to different electrode tracks) were made with glass micropipettes (5–10 MΩ). The signal from the electrode was amplified (gain 10000; bandpass 0.3–10 kHz,) then multiplexed in an audio monitor and a voltage window discriminator. The action potentials waveform and the corresponding TTL pulses generated by the discriminator were digitized (50 kHz sampling rate, Superscope, GW Instruments), visualized on-line and stored for off-line analyses. The pulses were sent to the acquisition board (PClab, PCL 720) of a laboratory microcomputer, which registered them with a 50 µsec resolution and provided on-line displays of the neuronal responses. Successive recording sites were separated by at least 100 µm in depth. At the end of the recording session (10–12 hours in duration), the animal was sacrificed by a lethal dose of pentobarbital (200 mg/kg).

### Histological Analyses

After each recording session, the brains were removed from the skull and placed in the fixative solution for two weeks. The brains were placed in a 30% sucrose solution in 0.1 M phosphate buffer for 3–4 days, then coronal serial sections of the brain were cut on a freezing microtome (50 µm thickness), mounted on glass slides, dried and counterstained with cresyl violet. The analysis of histological material was always done blind of the electrophysiological results. The sections were examined under several microscopic magnifications to find the electrode tracks corresponding to the tungsten electrodes. The depth coordinates read from the microdrive and determinations of the relative thickness of cortical layers in the guinea-pig ACx [32] were used to assign each recording to a cortical layer. Both in pilot experiments and in previous studies [29], [33] a good correspondence was found between the value read on the microdrive and the actual depth of small electrolytic lesions made via tungsten electrodes.

### Tuning Curves Determination

Each recorded cell was first tested with pure tone to determine its tuning curve at 70 dB. The cells included here were only those exhibiting reliable and stable tuning curves when tested twice with pure tones. The sound generating system used to deliver pure tone frequencies was the same as previously described [33]–[35]: Pure tones (100 ms, rise/fall time 5 ms, presented at 1 Hz) were generated by a remotely controlled wave analyzer (Hewlett-Packard model HP 8903B) and attenuated by a passive programmable attenuator (Wavetek, P557, maximal attenuation 127 dB), both controlled via an IEEE bus. Stimuli were delivered through a calibrated earphone (Beyer DT48) placed close to the ear canal. In situ calibration of the system was done with a probe tube using a sound level calibrator and a condenser microphone/preamplifier (Bruel and Kjaer models 4133 and 2639T) and a standard reference tone (1 kHz at 94 dB re 20 µPa) generated by the calibrator (B&K model 4230). The acoustic calibration provided a speaker output that could be corrected to ensure an almost flat frequency response (±6 dB from 0.5 kHz to 30 kHz) with minimal harmonic distortion (about 5%). The sound delivery system (the HP 8903B, the attenuators and the speaker) can deliver tones of 80 dB up to 20 kHz and of 70 dB up to 35 kHz. Harmonic distortion products were measured to be down about 50 dB from the fundamental.

### STRF Determination

Two sets of stimuli were used to compute STRFs: conspecific vocalizations and Dynamic Moving Ripples (DMRs). The spectrographic representation of vocalizations and DMRs were obtained using a gammatone filter bank constituted of 100 filters logarithmically spaced from 100 Hz to 22 kHz [36]. The energy envelope 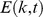
for each frequency band *k* was obtained by low-pass filtering at 50 Hz the half-wave rectified output of filter centered on *CF_k_*. The envelope was then resampled at 1 kHz using a cubic spline interpolation in order to match the PSTH bin size (1 ms).

**Figure 1:**
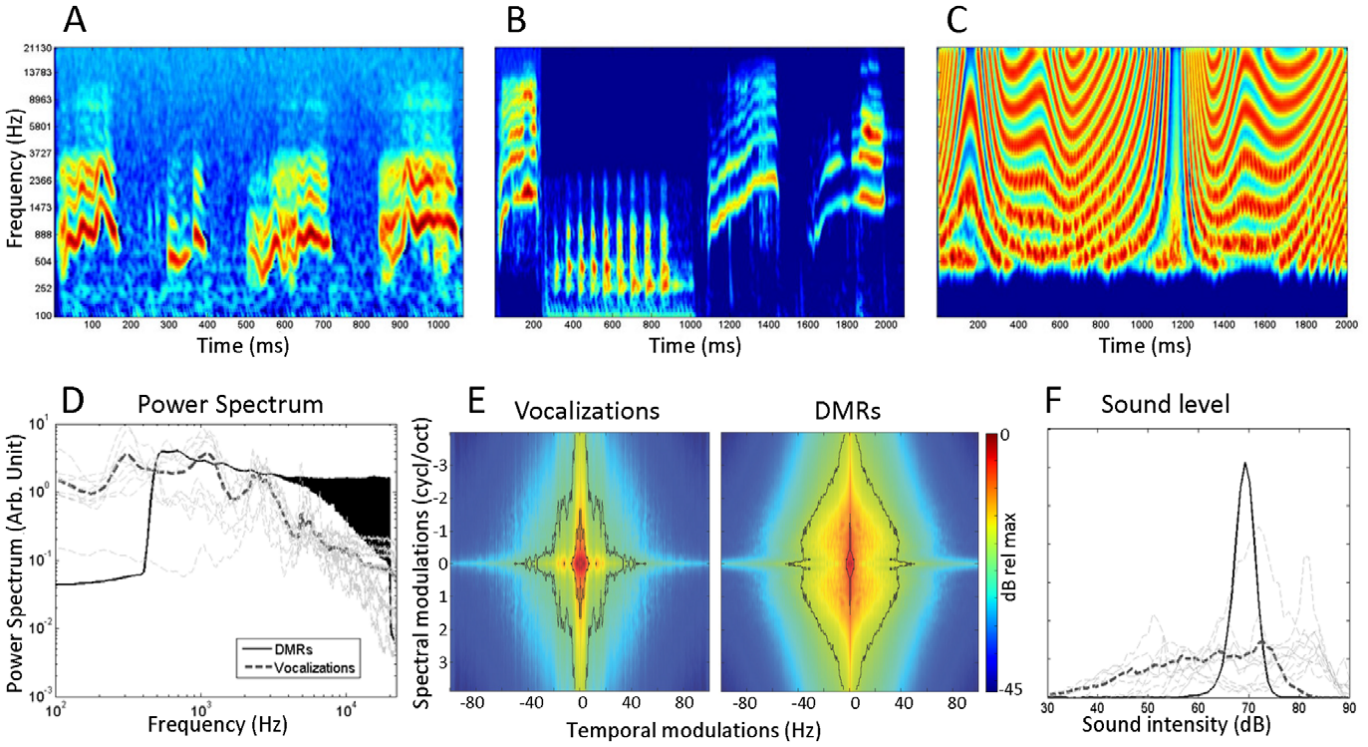
Statistics of the two sets of stimuli: Guinea-pig vocalizations and Dynamic Moving Ripples (DMRs). A. and B. Spectrograms of guinea-pig vocalizations. The chutter call is made of sound bursts in the medium frequency range (A). Whistle calls are high-pitched sounds rising in frequency whereas purr calls contain repetitions of low frequency sound bursts (B). C. Spectrogram of a DMR. All spectrograms are in logarithmic frequency scale. D. Average spectrum of our stimuli. The average spectrum of all DMRs is represented by the black line; the average spectrum of the vocalizations by the dashed dark grey line. The light grey line represents the spectrum of each vocalization file used. The vocalizations were selected to obtain a flat spectrum matching closely that of the DMRs. E. Modulation spectrum of the vocalizations and DMRs. As shown, both stimuli spanned the same range of temporal and spectral modulations. F. Distribution of sound intensity in 20 ms time bins calculated for both DMRs (filled-black line) and vocalizations (dashed dark grey line). Light grey lines show the distribution of sound intensity for each vocalization. The difference between the two distributions reflects the existence of large amplitude fluctuations through time in guinea-pig calls.

Natural stimuli consisted of 9 sound files composed of guinea-pig vocalizations from the guinea pig repertoire (purr, whistle and chutter – see Figure 1A–B). These vocalizations were not recorded from the set of guinea pigs used for the present study: they had been previously recorded from adult male guinea pigs of our colony (sampling rate 44 kHz, Sennheiser MD46 microphone, Sound Edit Software; see [30], [37]). On average, natural stimuli files were 2 sec long (ranging from 1 to 3.5 sec), their average sound level was set to 70 dB SPL and stimuli were presented 20 times at 0.5 Hz repetition rate. A set of vocalizations was selected such that their spectrum uniformly sampled the frequency range of [0.5–22 kHz]. The overall vocalization spectrum spanned frequencies from 0.1 to 22.5 kHz (Figure 1D).

We also computed the modulation spectrum of the vocalizations by performing a 2-D Fast Fourier Transform of the stimulus correlation matrix [38]. This matrix is obtained by cross-correlating the envelope 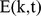
within each frequency band *k* to the envelope of all other frequency bands 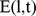 for 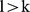. We display in Figure 1E the modulation power on a log-scale relative to the maximum. Two contour lines represent an attenuation of 25 and 50 dB from that maximum. It exhibited a star-like shape typical of natural sounds [20]. The power was concentrated on two branches of this star-like shape. The horizontal branch corresponds to temporal modulations with no spectral modulation (fluctuation of sound level in time), the vertical branch corresponds to low temporal modulations and power in spectral modulations (harmonic structure). Finally, we computed the modulation depth, 
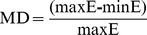
, and contrast,
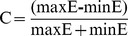
,where *E* denotes all samples of each stimuli envelope per frequency band. The modulation depth for the vocalization was 0.82 while the contrast was 0.69.

Dynamic moving ripples (DMR) were generated according to the method described by Escabi and Schreiner [10] but taking temporal and spectral modulation parameters in the appropriate range for cortical neurons. Briefly, the stimuli consist of a signal having temporal and spectral modulations varying randomly through time. The temporal modulations (variations of energy through time in each frequency band) were continuously varied from −50 to 50 Hz. Samples were uniformly drawn within that modulation ranges at 6 Hz and a cubic spline interpolation was used to resample the trajectory at 1 kHz. Spectral modulations (variations of energy along the frequency axis) varied from 0 to 2 cycl./oct. using the same procedure but sampling at 3 Hz. Note, however, that uniformly sampling these intervals before the cubic interpolation did not produce a uniform sampling in the modulation power spectrum of the DMRs (see Figure 1E). Indeed, since the instantaneous modulation followed a complex trajectory between the samples, the modulation power spectrum was biased with a trend similar to the vocalizations. To match the way the vocalization files were presented, we generated nine different DMRs of 2 seconds each at a sound level of 70 dB SPL. Each DMR file was repeated 20 times at a 0.5 Hz repetition rate. The procedure allows studying the trial-by-trial reliability of neuronal responses the same way as for the responses to vocalizations (see below). An example of DMR is presented in Figure 1C. The overall spectrum of the set of artificial sounds was flat over the range of frequencies considered. The spectro-temporal modulations of DMRs are displayed in Figure 1E. The modulation depth per frequency band of the DMRs was found to be inferior to that of the vocalizations (0.62) as was the contrast (0.45).

### Data Analysis

#### STRF calculation

For each cell, two STRFs were derived from the responses to the two sets of stimuli using a regularized reverse correlation technique performed with the STRFPak software [4]: a STRF_voc_ was obtained from the cell’s responses to all the vocalizations stimuli and a STRF_dmr_ was obtained from all the DMRs (except for the computations of the STRF predictive power as explained below). The STRF is a linear approximation of the stimulus-response function which relates the spectro-temporal representation of a stimulus to the neural response. The STRF is described as the linear kernel of the following convolution:
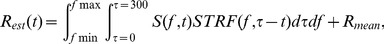
(1)where S(f,t) is the spectrographic representation of the stimulus (f_min_  = 0.5 kHz; f_max_  = 22 kHz), R_mean_ the mean firing rate of the observed response and R_est_ is the estimated firing rate giving a minimum mean square error. The STRF estimation is performed here using a reverse correlation technique. Indeed, defining the STRF as the linear system providing the least mean square error on the firing rate is equivalent to the following expression in the Fourier domain:
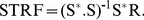
(2)The STRF can thus be estimated by multiplying the inverse of the power spectrum of the stimulus (S^*^S)^−1^ and the spectrum of the cross-correlation between the stimulus and the neuronal response S^*^R. This cross-correlation was obtained using the PSTH of the 20 repetitions binned with 1 ms bins. The PSTH were smoothed with a Hanning window of 20 ms half-width. In practice, singular value decomposition (SVD) is used when inverting the stimuli power spectrum. SVD allows to retain only the significant components of the stimuli power spectrum and a tolerance factor is chosen to determine the level at which these components are considered significant. This regularization procedure implements a smoothing constraint on the STRF (modulation power mainly in the low frequency region) similar to procedures applied in other studies [22]. The regularization parameter providing the best correlation coefficient (CC) between the measured and predicted rate was chosen when analyzing our cortical recordings. As did other groups when using repetitions of brief broadband stimuli [39], we removed the neuronal activity occurring in the first 50 ms of each DMR to exclude onset responses from the STRFs computation.

We computed STRF significance using a bootstrap algorithm [8], [9]. Bootstrap estimates of the STRF were computed from 100 different random combinations with replacement of pairs of stimuli and evoked responses. Each STRF obtained was used to compute a standard deviation per pixel. We used the average variance over all pixels of the STRF to define two significance contours at values above 3 and above 5 times the standard deviation, σ (respectively black and white lines in the figures showing individual examples). The significance contours were then used when assessing the STRF shape differences. We discarded from our analysis the STRFs of cells which met at least two of the following criteria: (a) few responses on their raster, (b) small and scattered significant zones in the STRF which varied with the regularization parameter value and (c) very poor STRF predictions (measured by CC<.05, see below). About 30–40% of the cells were discarded of the subsequent analyses (see the percentages in the 2^nd^ paragraph of the Results section).

#### Goodness of fit for the STRF prediction (CC)

The STRF predictions were assessed using the correlation coefficient (CC) between the predicted and measured response. The correlation coefficient is given by:
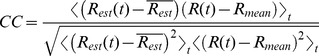
(3)where 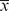 denotes averaging over the stimulus set and 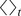 averaging over time. Predicted responses were obtained by the convolution of the STRF with a test stimulus not used for the STRF computation. For instance, using vocalizations #1 to 8, we computed a STRF which was then convolved with the spectrogram of vocalization #9. The result of this convolution gave the prediction to vocalization #9 which was compared to a smoothed measured response. This smoothed response corresponds to the PSTH from 10 trials smoothed using a Hanning window with 20 ms half-width. By repeating this procedure to all vocalizations (respectively DMRs), we computed an average CC_voc_ (respectively CC_dmr_).

The CC_voc_ and CC_dmr_ constitute a goodness of fit measure which is affected by the inter-trial variability of the measured response. Since the PSTH is a noisy estimate of the neuron’s response, there is a maximal value that predictions can reach (i.e. CC_voc_<1 and CC_dmr_<1). That value depends on the level of noise in the PSTH. To account for such effects, we compared the CC_voc_ and CC_dmr_ to the level of inter-trial variability. We assessed this level of inter-trial variability using a correlation coefficient between two smoothed PSTHs estimates (cf previous paragraph) obtained from half of the 20 trials (CC_psth-psth_). In order to obtain a correct estimate of CC_psth-psth_, the procedure was repeated 10 times for different random permutations. Finally, we evaluated which STRF predictions significantly captured the cell’s response signal (i.e. the noise-free part of the neuron’s response) by performing a paired t-test between CC_voc_ (respectively CC_dmr_) and CC_psth-psth_ for 10 random permutations of PSTH estimates.

#### Comparison between STRF derived from vocalizations and from DMRs

The comparison between STRFs obtained from vocalizations and DMRs was performed on both STRF predictive power and STRF differences in shape. The correlation coefficient (see above) was also computed between stimulus ensembles. These predictive powers are denoted CC_voc2dmr_ when using the STRF_voc_ to predict responses to DMR and CC_dmr2voc_ when using the STRF_dmr_ to predict the responses to vocalizations.

The differences in shape for the STRF were quantified using the similarity index (SI) previously introduced by [10]. This index is given by:
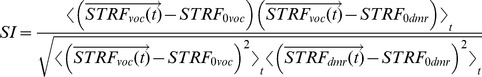
(4)where 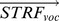 (respectively 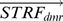) is a vector version of the STRF_voc_ (respectively STRF_dmr_) and *STRF*_0*voc*_ (respectively *STRF*_0*dmr*_) is the mean value of 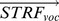 (respectively 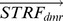). The SI corresponds to a correlation coefficient computed between the two STRFs reshaped into vectors. Pixels outside the significant contours were set to zero for this analysis. The similarity index approaching a value of 1 indicates that the STRFs have identical shapes; a value close to 0 indicates that the shapes are totally different and a value of −1 indicates that regions of excitation are replaced by regions of inhibition and vice-versa.

#### Comparison between parameters derived from STRF and from classical tuning curves

STRFs were used to measure cell’s response properties such as best frequency (BF), bandwidth (BW) and latency of the response. The BF was defined as the frequency for which the value obtained in the STRF was maximum, the BW as the frequency range over which positive STRF values were significant and latency as the time at which the STRF reached its maximum value. These parameters were compared to those obtained from classical tuning curves and compared between STRF_voc_ and STRF_dmr_.

#### Linear spiking model of neural processing

To estimate the putative causes of the differences between STRF_voc_ and STRF_dmr_, (assessed by the similarity index SI), we built a neural model in which processing was set linear and similar for DMRs and vocalizations. A two-stage model was used to generate a set of artificial STRFs. This model was constituted of (1) a linear filter stage where the stimulus was convolved to an artificial STRF (STRF_art_), followed by (2) a non-homogenous Poisson process (NHPP) used to mimic the discrete spiking events on a single trial basis. Then, from these surrogate spike trains, we computed the STRF (denoted STRF* in the following) as we did from the real spike trains. We used the same PSTH smoothing parameters, significant contours and method for choosing the tolerance factor as for the real data. Since the NHPP model produces a firing rate depending linearly on the spectro-temporal properties of the stimuli, the estimated STRF* obtained from the regularized reverse correlation technique should only differ from the STRF_art_ due to the limited statistics available in our experimental paradigm (i.e. the limited number of stimulus presentations and the low firing rate of cortical neurons under anesthesia, see below).

To match with the physiological data, we produced 42 artificial STRF_art_ and generated 20 responses for each vocalization and DMR. The STRF_art_ were constituted by excitatory and inhibitory zones in the temporal and spectral domains. To produce the zones, we used either a Gaussian function (purely excitatory STRF_art_) or the first derivative of a Gaussian (STRF_art_ with lateral inhibition on one side) or its second derivative (STRF_art_ with surround inhibition). More precisely, STRF_art_ were set according to 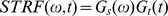, where 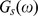 (respectively 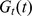) denotes the STRF shape in the spectral (respectively temporal) domain.

As the NHPP model was built in order to control for putative biases in the stimulus ensembles, it was necessary to match it as closely as possible to the cortical data. In the spectral domain, 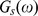 was set to match the range of the center frequencies observed in our data and its standard deviations to match the measured bandwidths. In the temporal domain, the latencies of the artificial STRFs were chosen in the range observed in our population. Considering the NHPP model response strength and reliability, we set its average firing rate to match the mean evoked firing rate of the population of the recorded cells. Moreover, the spike timing reliability (as indexed by the Rcorr, see below) was computed and found to be similar for the cortical data and the NHPP model (unpaired t-test for vocalizations, p = 0.32; for DMRs, p = 0.12).

So far, the artificial STRF were described by: 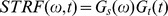, i.e. as the product of a function of space and a function of time. Such artificial STRFs are called separable but it should be noted that not all STRFs in our data can be expressed in a separable form. Hence, as a subsequent control, we considered the case of unseparable artificial STRFs. In order to produce unseparable STRF_art_, we rotated the separable kernel by an angle □ in the spectro-temporal plane. The angle was chosen to be uniformly distributed between [−π/4, π/4]. This procedure is sufficient to make the STRF unseparable and assess the effect of separability on the bias of the estimation procedure. In the Result section, unless explicitly mentioned, the results are only presented for separable STRF_art_.

#### Spike-timing reliability of neuronal responses

As in previous studies [30], [40], we evaluated the trial-to-trial spike-timing reliability to a given stimuli using the Rcorr index introduced by Schreiber et al. [41]. This index measures the average correlation across trials (i.e. between the spike trains obtained from several repetitions of the same stimulus). This correlation is given by a scalar product between pairs of trials, the result being divided by the norms of the two trials. Each spike train 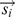, used for the scalar product, is given by a vector of zeros and ones convolved by a Gaussian window of different standard deviations σ. Therefore, the correlation measure Rcorr is given by:
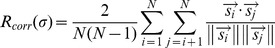
(5)in which *N* is the number of stimulus presentations. The value of Rcorr typically increases as a function of the smoothing window’s width σ. This is due to the progressive removal of any temporal differences between pairs of spike trains when the size of the smoothing window gets larger. Window sizes ranging from 1 to 90 ms were analyzed. The results presented here are for σ  = 20 ms. A possible bias in the Rcorr statistic can come from the stimuli length [30]. To avoid this bias, Rcorr were computed using a sliding window of 200 ms and the averaged Rcorr was then kept as the final result.

#### Effects of bursts of action potentials on STRF computation

Bursts are groups of action potentials emitted with short inter-spike intervals (<5 ms). They constitute one of the many nonlinear neuronal mechanisms that can alter the procedure of STRFs computation. Indeed, the nonlinear dynamics typical of bursting behavior [42]–[44] is very different from a linear filter model such as the STRF. For instance, thalamic low-threshold bursts have been shown to generate different STRFs than single spikes [45]. To assess the importance of this nonlinearity, the number of bursting events was determined using a detection procedure applied in previous studies [46]–[51]. Here, bursts were defined as groups of action potentials (APs) in which the two first APs are separated by 5 ms or less (with the possibility that the two last APs are separated by 10 ms when the burst is made of more than 2 spikes). These criteria have been mainly applied at the thalamic level, but they also successfully detected the increase in bursts proportion in auditory cortex at switch from waking to slow wave sleep [33]. Although this procedure differs from non-parametric ones based on deviation from a Poisson process [52], we chose it because it is based upon physiological (but more conservative) criteria. The percentage of bursting events was defined as:
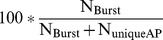
.

For a subset of cells exhibiting at least 100 bursts over all trials in each stimulus set (i.e., over the 20 repetitions of the 9 DMRs or 9 vocalization files), we split each evoked spike train into two components: the single APs component from which we computed a STRF_AP_ and the burst component from which we computed a STRF_Burst_.

## Results

Only cells exhibiting stable action potential waveforms during the entire recording session (≈60 min) were included in the present study. Ninety two cells, all exhibiting robust responses to pure tones, were recorded in the primary auditory cortex of adult guinea-pigs. Two to thirteen cells were collected from each animal (mean 9 cells/animal). Cells were recorded from 200 to 2050 µm below pia and, based on the laminar analyses performed by Wallace and Palmer [32], each cell was assigned to a cortical layer. Using pure tones, the characteristic frequency (CF) was between 0.7 and 20 kHz and the threshold between 0 to 60 dB SPL. During the tuning curve determination, spontaneous activity ranged from 0.01 to 3.4 spikes/sec (median 0.42; mean±sem 0.81±1.12) and at 20 dB above threshold the responses at the best frequency ranged from 10.2 to 65.3 spikes/sec (median 15.1; mean±sem 20.5±8.2).

### Characterization of the Cells with Significant STRFs

**Figure 2:**
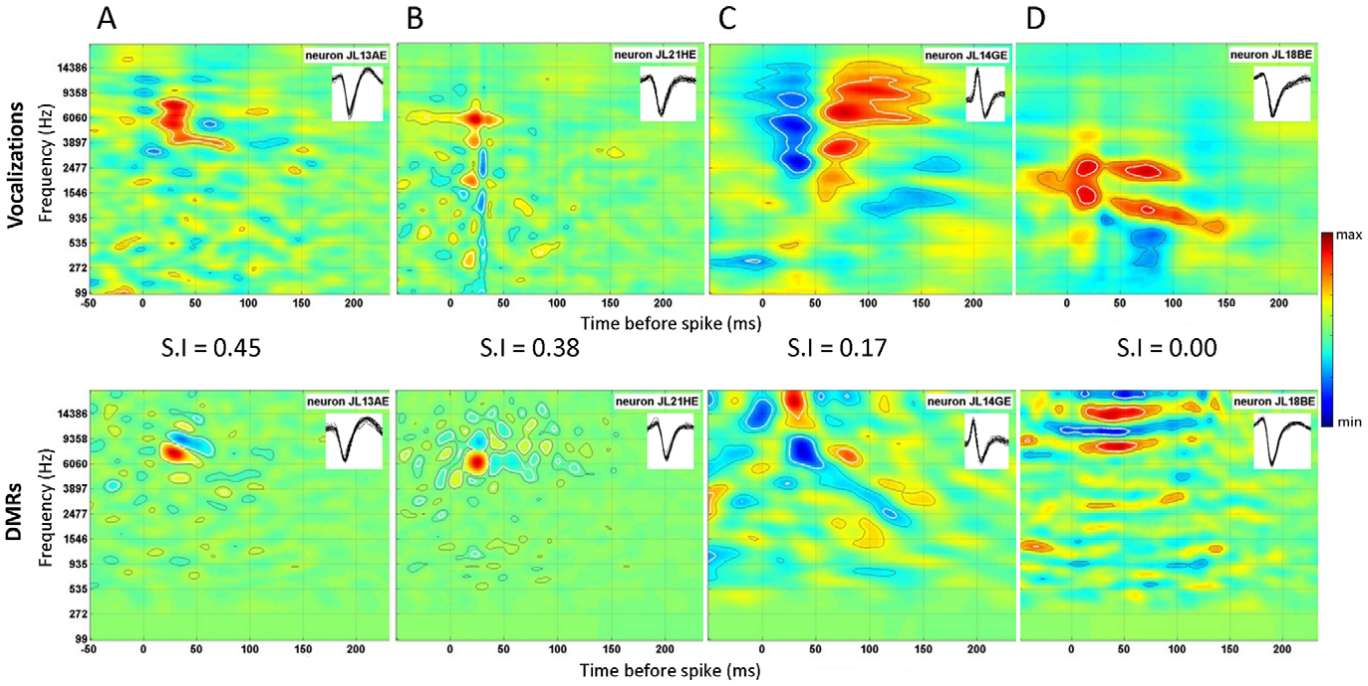
Individual examples for four neurons exhibiting either similar (A. and B.) or different (C. and D.) STRFs derived from responses to vocalizations (top row) and responses to dynamic moving ripples (DMR, bottom row). For each STRF, the color code represents “excitation” in red and “inhibition” in blue, units are in spikes/(sec^2^*dB). In A. and B., the neurons display relatively similar STRFs with vocalizations and DMRs: The best frequency (BF) was similar with vocalizations and DMRs (6.5 kHz in A and B) and the excitation area was circumscribed to the same frequency range, despite some differences in the overall shape. Thus, the similarity index (SI, indicated between the two rows) was relatively high (0.45 and 0.38). In C. and D. the neurons display quite different STRFs estimated from responses to vocalizations and DMRs; in particular, the BF, the shape and the frequency range of the excitatory area differed. Therefore, the values of the SI are low (0.17 and 0.00). Insets in each STRF show the AP waveform during presentation of the stimuli.

From the responses collected at presentation of vocalizations, STRFs exhibiting significant zones (see Methods) were obtained for 62% (57/92) of the cells. From the responses collected at presentation of DMR, STRFs exhibiting significant zones were obtained for 68% (63/92) of the cells. Figure 2 displays examples of four STRFs obtained in both conditions. In all figures, black and white lines indicate significance contours at 3 and 5 σ, respectively (red indicates “excitatory areas” and blue indicates “inhibitory areas”).

First, we looked for potential differences between cells whose responses could be fitted with significant STRFs (with DMR and/or with vocalizations) compared to cells which could not. There was no difference between these two cell populations in terms of cortical depth, breadth of tuning and response latency when tested with pure tones (see Table 1). Cells showing significant STRF_voc_ had a higher spontaneous rate than those which did not, but spontaneous activity was not a critical factor because such effect was not observed for cells showing significant STRF_dmr_. Although cells showing no STRF_dmr_ had a higher percentage of evoked bursts in their responses, this was not the case for the STRF_voc_. In contrast, both for the responses to DMRs and to vocalizations, the strength of evoked responses and their temporal reliability were higher for cells whose responses allowed the estimation of a significant STRF compared to cells which did not (see Table 1).

**Table 1:** Characteristics of the cells exhibiting significant STRF compared with those exhibiting no significant STRF using vocalizations and Dynamic Moving Ripples (DMRs).

	Responses to vocalizations			Responses to DMRs		
	STRF (n = 57)	No STRF (n = 36)	p value	STRF (n = 63)	No STRF (n = 29)	Stat diff
Mean Depth (µm)	1126	1130	p = 0.32	1129	1007	p = 0.23
(range µm)	350–2150	200–1970		270–2150	200–1950	
Tuning width						
Q20 dB	1.82	1.78	p = 0.43	1.70	1.66	p = 0.85
Latency						
Mean (ms)	35.6	34.1	p = 0.63	36.2	32.4	p = 0.23
sem (ms)	19.3	18.9	p = 0.82	19.1	19.2	p = 0.96
Spontaneous	2.72	1.29	**p = 0.01**	2.13	2.34	p = 0.78
Evoked	5.71	2.97	**p = 0.001**	5.16	2.92	**p = 0.04**
% Evoked bursts	7.99	5.95	p = 0.12	5.91	10.70	**p = 0.001**
Rcorr	0.46	0.39	**p = 0.01**	0.43	0.32	**p = 0.002**

Unpaired t-tests were used to determine if the parameters differed between these 2 types of cells.

**Figure 3:**
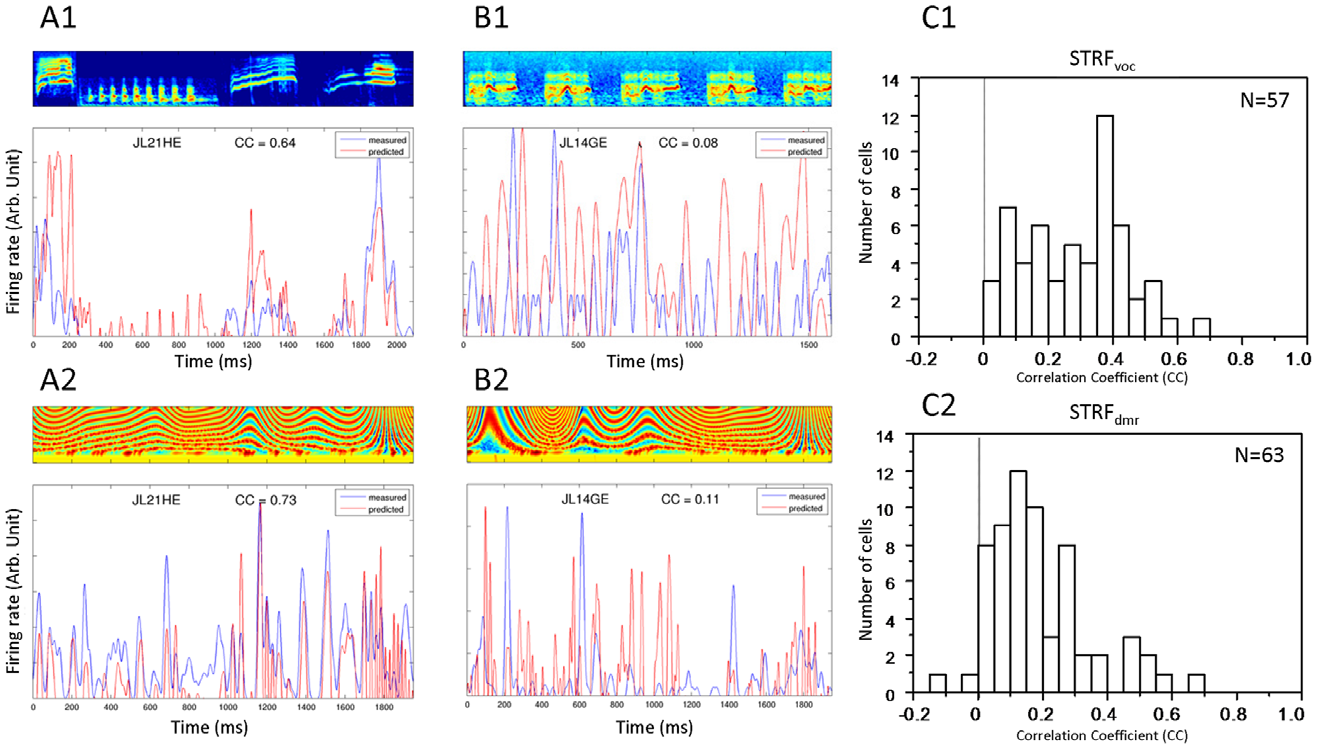
Predictions between measured responses and predicted responses based on the STRF_voc_ (top, A and C) and on the STRF_dmr_ (bottom, B and D) quantified by the Correlation Coefficients (CC). A–B. Individual examples showing the actual measured responses for a neuron presenting good predictions (JL21HE; left panel) and a neuron presenting poor predictions (JL14GE, middle panel). The top row shows the responses to vocalizations corresponding to spectrograms displayed on the top with the measured responses in blue and the predicted responses in red. The bottom row shows the responses to DMR corresponding to spectrograms displayed on the top (blue curve, measured responses; red curve, predicted responses). C1–C2. Distributions of the CC values for the STRF_voc_ (A) and for the STRF_dmr_ (B). The mean value of 0.29 obtained for the STRF_voc_ is significantly higher than the mean value obtained for the STRF_dmr_ (0.19). The CC values presented here are for STRF_voc_ tested on vocalization responses and for STRF_dmr_ tested on DMR responses.

Second, we investigated the STRFs’ predictive power and compared it to the inter-trial variability. The predictive power of STRF_voc_ and STRF_dmr_ was quantified by the correlation coefficient between the actual PSTH and the predicted PSTH from the STRF (CC_voc_ and CC_dmr_; see Methods). Two individual examples are presented in Figure 3A–3B, one with a high (JL21HE, Figure 3A1–A2) and the other with a low (JL14GE, Figure 3B1–B2) predictive power. The distributions of the predictive power measures CC_voc_ and CC_dmr_ are presented in Figure 3C for all cells with significant STRF_voc_ (C1) and/or STRF_dmr_ (C2). They significantly differed (χ2 = 24.18; p = 0.04) and so did their mean values (0.288 for STRF_voc_ vs. 0.191 for STRF_dmr_; unpaired t-test, p<0.01), indicating that for the cell population under study, responses to vocalizations are better predicted by STRF_voc_ than responses to DMRs are predicted by STRF_dmr_.

**Figure 4:**
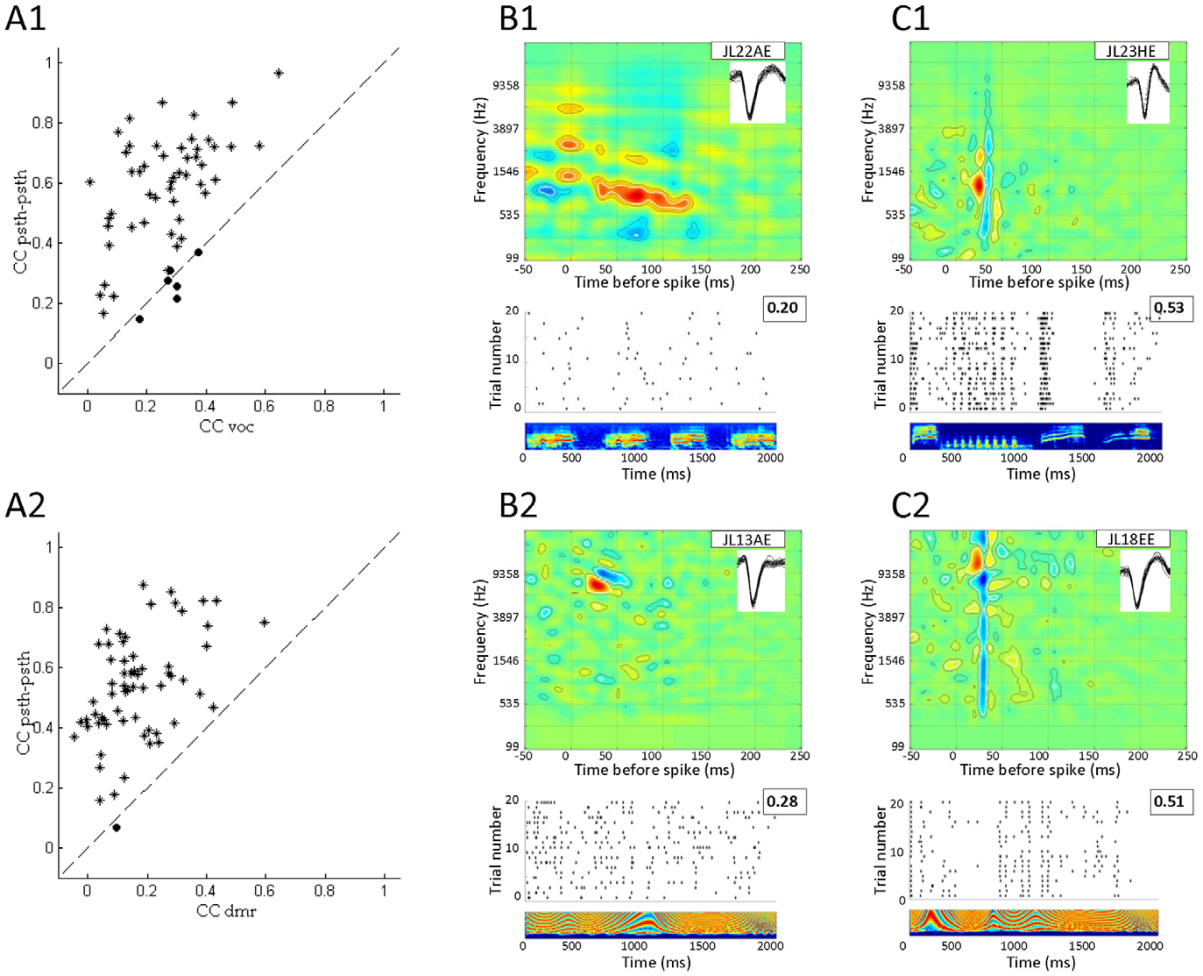
Inter-trial variability. A. Comparison between the inter-trial variability and the predictive power. The inter-trial variability computed with the Correlation Coefficient between PSTH (CC_psth-psth_) is compared for each cell to the predictive power for the vocalizations (A1) and the DMR (A2). In most of the cases (black stars in A1 and A2), CC_psth-psth_ is significantly higher (paired t-test, p<.05) than CC_voc_ (A1) or CC_dmr_ (A2). In very few cases (black dots), CC_psth-psth_ is not significantly different than CC_voc_ or CC_dmr_. B–C. Examples of four neurons showing either a low (B) or a high (C) spike timing reliability. For each plot, the neuron’s STRF (top) is shown, together with the raster plot (middle) of 20 responses to the stimulus for which the spectrogram (frequency vs. time) is represented on the bottom. Values inserted along the raster plots are the values of the trial-by-trial spike timing reliability as computed with the Rcorr. B1 and B2 show responses with a low spike-timing reliability, whereas C1 and C2 show responses with a high spike-timing reliability. Insets in each STRF show the AP waveforms. STRF units and scale are the same as in Figure 2.

We then investigated how linear the stimulus-response relationship is (for the vocalizations and the DMRs) by comparing the predictive power (CC_voc_ and CC_dmr_) to the inter-trial variability (CC_psth-psth_). The latter was computed using the correlation coefficientapplied on the cells’ PSTH splitted into two sets of 10 trials (see Methods). For a large majority of cells (51/57 for vocalizations and 62/63 for DMRs), both the CC_voc_ and CC_dmr_ were significantly smaller (paired t-test, p<0.05) than the CC_psth-psth_ (see Figure 4A1–A2). Figure 4A1 shows that for almost all cells showing significant STRF_voc_, the CC_psth-psth_ is high and higher than the CC_voc_. On average over the whole population, the CC_voc_ was 2.85 times larger than the CC_psth-psth_. For the DMRs, the CC_psth-psth_ was on average 4.07 times larger than the CC_dmr_ (Figure 4A2). These results show that a low predictive power cannot be solely attributed to the inter-trial variability. Indeed, for the cells in the upper left part of Figures 4A1 and 4A2, the cells responses are highly reproducible (high CC_psth-psth_ value), but still the responses are poorly fitted by the STRF model, resulting in a low CC_voc_ (or CC_dmr_) value. Noteworthy, the CC_psth-psth_ did not significantly differ between the two stimulus sets (CC_psth-psth_ = 0.55 for the vocalizations and CC_psth-psth_ = 0.53 for the DMR; unpaired t-test, p = 0.43), indicating that the inter-trial variability was similar for vocalizations and DMR.

### Spike-timing Reliability is Independent of the Stimulus Set

Over the 20 repetitions of each vocalization and DMR file, the spike trains can exhibit either a low (Figure 4B1–B2) or a high (Figure 4C1–C2) trial-to-trial temporal reliability. For each cell, we quantified the spike timing reliability at presentation of natural and artificial stimuli by the Rcorr index ([41], see Methods). The mean Rcorr values were not significantly different when quantified during the presentation of DMRs and of vocalizations (Rcorr_dmr_  = 0.44, Rcorr_voc_  = 0.46; paired t-test, p = 0.11). These values were as high (or higher) than those obtained in our previous studies [30], [40]. As indicated in Table 1, the Rcorr index was higher for responses which could be predicted by significant STRFs than for responses which could not, and this hold true for both STRF_voc_ and STRF_dmr_. A correlation was observed between Rcorr_voc_ and the values of CC_voc_ for the responses to vocalizations (r = 0.37, p<0.05) but not between Rcorr_dmr_ and the values of CC_dmr_ (r = 0.22, p = 0.17). This can be explained given that the CC_voc_ and the CC_dmr_ were not correlated (r = 0.08, p = 0.6, - see below, *Quantification using the predictive power*) despite the correlation between Rcorr_voc_ and Rcorr_dmr_ (r = 0.4, p<0.01). Thus, spike-timing reliability (indexed by Rcorr) is independent of the stimulus set.

### Comparison between STRFs Obtained from Natural and Artificial Stimuli

The responses of forty-two neurons (45%) allowed estimation of significant STRFs from both vocalizations and DMRs. Unless explicitly specified, all subsequent comparisons will focus on this population of 42 cells. For these cells, the strength of evoked responses did not significantly differ between the DMRs and the vocalizations (paired t-test; p = 0.82). Figures 2A and 2B depict two STRFs showing similarities when computed from vocalizations and DMR: despite slight differences in the shape of the excitatory areas, these areas are in the same frequency range. However, in many cases the STRF_voc_ and STRF_dmr_ exhibited important differences. Figures 2C and 2D show two examples of cells exhibiting important mismatches between their STRF_voc_ and STRF_dmr_: Not only the shapes of the excitatory and inhibitory areas differ but they are also located in different frequency ranges. The differences between STRF_voc_ and STRF_dmr_ were quantified by classical measurements (BF, bandwidth and latency; see below), a similarity index and their predictive power.

#### Quantification using classical parameters

**Figure 5:**
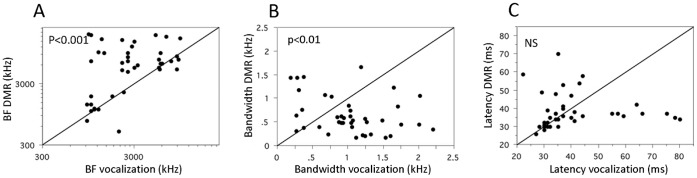
Comparison between the STRF parameters derived from the STRF_voc_ and the STRF_dmr_. A. Scattergram showing the values of the BF derived from the STRF_voc_ (abscissa) against the values of the BF derived from the STRF_dmr_ (ordinates). For half of the cases, the values are similar (dots around the diagonal line) whereas for the other half, the values derived from the STRF_dmr_ were higher than those derived from the STRF_voc_. B. Scattergram showing the bandwidth values derived from the STRF_voc_ (abscissa) against the bandwidth value derived from the STRF_dmr_ (ordinates). In many cases, the values were lower with STRF_voc_ indicating a larger bandwidth of excitatory responses when tested with vocalizations. C. Scattergram showing the latency values derived from the STRF_voc_ (abscissa) against the bandwidth value derived from the STRF_dmr_ (ordinates). The latencies of the excitatory responses were often similar but, in some cases, they were lower with DMRs than with vocalizations. STRF units and scale are the same as in Figure 2.

Best frequencies, bandwidths and latencies of the excitatory area extracted from STRF_voc_ were compared with those obtained from the STRF_dmr_. Important differences were noted and there was no significant correlation between the parameters derived from both types of STRFs (lowest p value p = 0.15 for the BF values). For example, the BFs derived from STRF_dmr_ and from STRF_voc_ rarely matched (paired t-test, p<0.001; Figure 5A). As shown in Figures 5B and 5C, discrepancies between STRF_voc_ and STRF_dmr_ also exist in terms of bandwidth of excitatory area (paired t-test, p<0.01) and in some cases in terms of response latencies (p = 0.17). The values of the bandwidths derived from STRF_voc_ and STRF_dmr_ were higher than those obtained from pure tones (p<0.01 in both cases) and the response latencies were smaller (p<0.001 in both cases).

Interestingly, the BFs derived from the tuning curves usually matched the BFs derived from the STRF_dmr_ (paired t-test, p = 0.69) but did not match the values derived from the STRF_voc_ (paired t-test, p<0.0001, see Figure S1). This could suggest that STRF_dmr_ better capture the classical receptive field properties. For each cell, we computed the coordinate of the STRF_dmr_ maxima (ie, a point in the time-frequency domain corresponding to the latency and BF of the cell as extracted from the STRF_dmr_) and looked whether at this point, the STRF_voc_ was excitatory or not. This was true for 21/42 cells (50%) but for 19/42 cells (45%) the STRF_voc_ was not significantly excitatory at the point (defined by the BF and the latency) where the STRF_dmr_ was maximum (the two other STRFs were inhibitory at this point). Performing the reverse analysis, we found that for 17/42 cells (44%), the STRF_dmr_ was excitatory at the location where the STRF_voc_ was maximum, but for 21/42 (50%) of the cells, the STRF_dmr_ was not significantly excitatory. Finally, note that only 13/42 (31%) cells showed a consistent excitatory STRF in both analyses, meaning that for 69% of the cells, either the maximum of STRF_voc_ was unrelated to features in the DMR condition or the STRF_dmr_ was unrelated to features in the vocalization.

In order to emphasize that the differences between STRF_voc_ and STRF_dmr_ were not only a consequence of the differences between artificial and natural stimuli, we built a linear spiking model and simulated its responses to the two sets of stimuli (see Methods). We then computed STRF*_voc_ and STRF*_dmr_ for this linear spiking model, and extracted from these STRFs* the best frequency, the bandwidth and the latency. As expected, all these parameters were highly correlated between STRF*_voc_ and STRF*_dmr_ (Best Frequency: r = 0.72, p<0.001; bandwidth: r = 0.48, p<0.01; latency: r = 0.34, p<0.05). The bandwidths and latencies were not significantly different (paired t-test, p = 0.88 for the bandwidth and p = 0.51 for the latencies) between STRF*_voc_ and STRF*_dmr_, however, the BF were slightly, but significantly smaller for the vocalizations than for the DMR (mean BF = 3.4 for the vocalizations, mean BF = 3.5 for the DMR, paired t-test, p = 0.03).

#### Quantification using the Similarity Index (SI)

**Figure 6:**
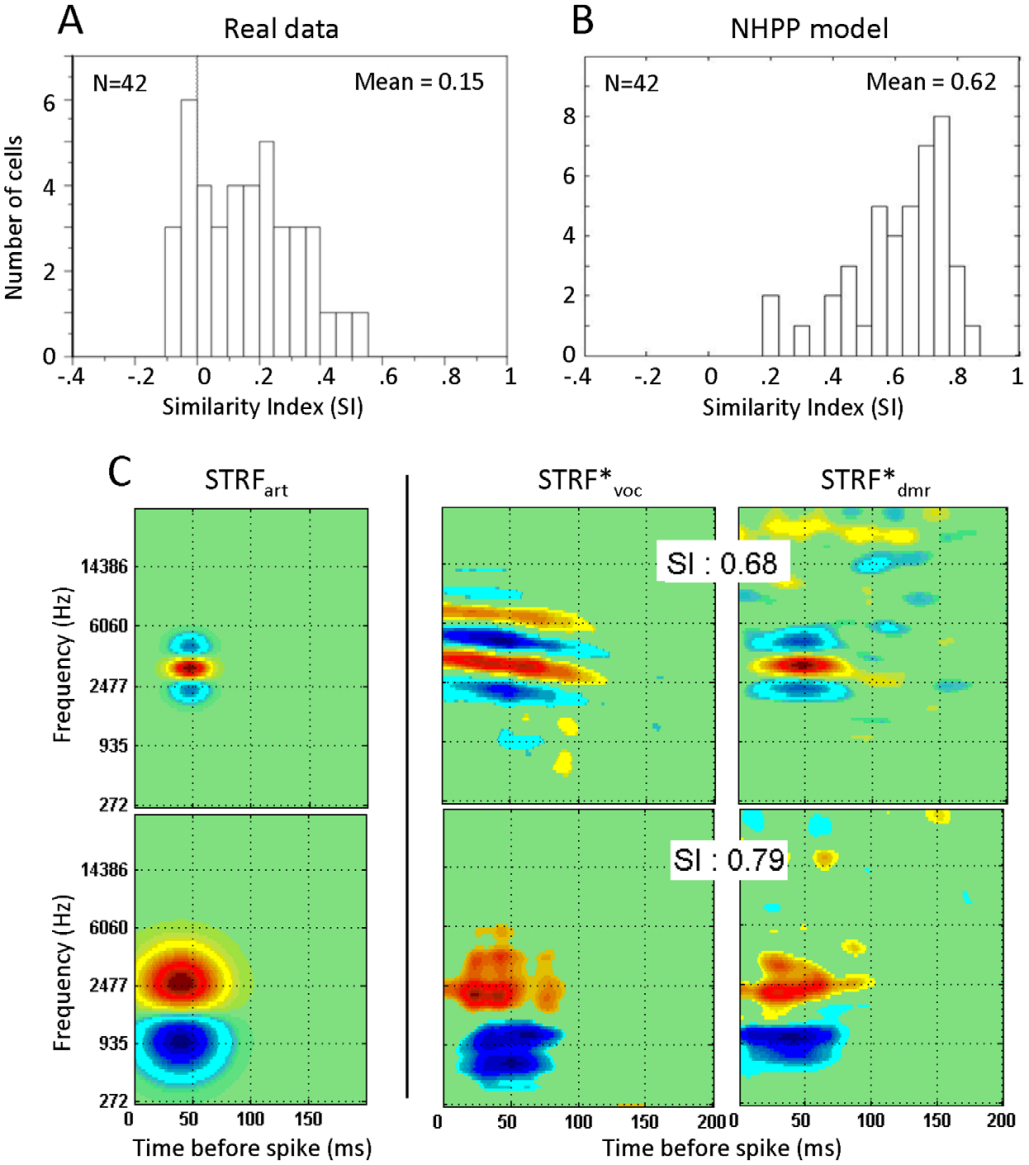
Comparisons between the similarity index (SI) obtained with the real and artificial neurons. A. Distribution of SI for the 42 neurons allowing for a paired-comparison between STRF_voc_ and STRF_dmr_. The mean SI of 0.15 is low and reflects important differences between the two types of STRFs. B. Distribution of SI for 42 STRF*s generated from the NHPP model responses. The mean SI of 0.62 reflects that STRF*s from vocalizations and DMRs show numerous similarities when calculated from the NHPP model’s responses. C. Two examples of STRF_art_ (left) used to generate surrogate responses with the NHPP model and their associated estimated STRF*_voc_ (middle) and STRF*_dmr_ (right). The first example (top) displays a narrow excitatory zone surrounded by two inhibitory side bands. The second STRF_art_ (bottom) is wider in spectral extent and has an inhibitory zone only in the low frequencies. Estimated STRF*s obtained from both sets of stimuli are quite similar with high SI values (0.68 and 0.79). Hence, the low SI values obtained from real data (A) cannot be attributed solely to differences in the statistics of our stimuli. STRF units and scale are the same as in Figure 2.

The SI quantifies the similarity between STRF_voc_ and STRF_dmr_ by taking into account the shape, frequency range and strength of significant excitatory and inhibitory areas [10]. The mean SI value was 0.15 (range –0.1 to 0.5) and its distribution is biased toward low values (Figure 6A). As for the classical tuning curve parameters, we computed the similarity between the STRFs* produced by the linear spiking model. If the differences between STRFs that we observed in the real data were only due to the stimuli, low SI values should also be obtained with the model. Actually, with the model, we obtained a relatively high mean SI value of 0.62 (range 0.27 to 0.90; Figure 6B). Slightly smaller values of SI were obtained using unseparable STRF_art_ (mean = 0.52). The distribution of SI values obtained using the linear spiking model on separable STRF_art_ clearly differs from that of the real data (χ2 = 63.5; p<0.0001). Figure 6C show examples of the artificial STRF (STRF_art_ used to generate surrogate spike trains), STRF*_voc_ and STRF*_dmr_ estimated from the NHPP model responses. The higher SI values obtained with the model compared to the real data indicate that the differences between STRF_voc_ and STRF_dmr_ cannot simply be accounted for by differences in the stimulus statistics. Note that (as mentioned in the Methods section), the NHPP model matched the physiological data with regards to the STRF characteristics (BF, BW and latency) but also in terms of spiking response (mean firing rate and spike timing reliability). Indeed, the Rcorr did not differ between cortical and surrogate spike trains (unpaired t-test for vocalizations, p = 0.32; for DMRs, p = 0.12). The inter-trial variability (as indexed by the CC_psth-psth_) was lower for the surrogate responses than for the cortical responses (unpaired t-test for vocalizations p<0.001; for DMRs, p<0.001) indicating that, with surrogate responses which are noisier than the real data, the NHPP model still produces STRF*s that show more similarity between stimuli than the cortical data. This emphasizes that the observed differences between cortical STRF_voc_ and STRF_dmr_ originate from other factors than the acoustic differences between stimuli.

#### Quantification using the predictive power

As described for the entire population, for the 42 cells whose STRF_dmr_ and STRF_voc_ were both significant_,_ the values obtained for the CC_voc_ (measuring the prediction of the STRF_voc_ on a new vocalization) were significantly higher than the CC_dmr_ (0.30 vs. 0.17; paired t-test, p<0.0001). Interestingly, this was also the case for the NHPP model (0.37 vs. 0.26; p<0.0001). To assess whether this better predictability for the vocalizations originated from a bias in the estimation procedure, we computed the CC_voc_ and CC_dmr_ when STRF* were estimated at equal regularization values for both stimulus set. For all tolerance values used, the NHPP predictions were significantly higher for vocalizations than for DMRs (paired t-test; p<0.0001).

**Figure 7:**
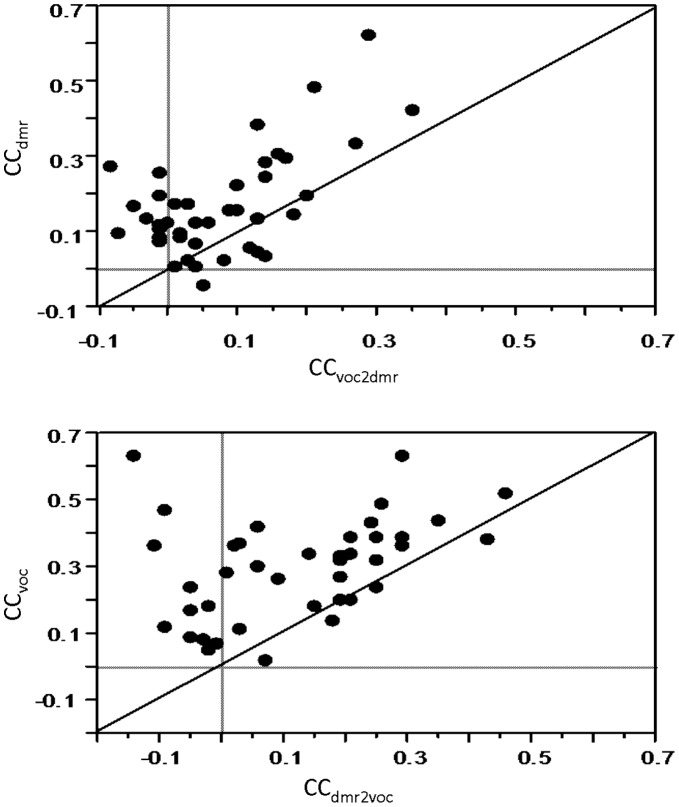
Across stimuli STRF predictions. Top: Scattergram showing the CC values when STRF_dmr_ are used to predict responses to DMR (CC_dmr_, ordinates) against CC values when STRF_voc_ is used to predict responses to DMR (_CCvoc2dmr_, abscissa). Bottom: Scattergram showing the CC values when STRF_voc_ are used to predict responses to vocalizations (CC_voc_, ordinates) against CC values when STRF_dmr_ is used to predict responses to vocalizations (CC_dmr2voc_, abscissa). In both cases, the large majority of values are higher when the same stimulus set is used for computing the STRF and for predicting the response (dots are mainly above the diagonal line).

Since the predictive power values spanned a large range for both DMRs and vocalizations (−0.2 to 0.63), it could be suspected that cells with a low CC_dmr_ also show a low CC_voc_. This was not the case: there was no correlation between CC_voc_ and CC_dmr_ (r = 0.08, p = 0.6). Not surprisingly, when the STRF derived from one set of stimuli was used to predict responses to the other set, the predictive power decreased significantly (Figure 7). This holds true both when using the STRF_voc_ to predict responses to DMRs (CC_dmr_  = 0.17 vs. CC_voc2dmr_  = 0.08; paired t-test, p<0.0001) and when using the STRF_dmr_ to predict responses to vocalizations (CC_voc_  = 0.30 vs. CC_dmr2voc_  = 0.12; paired t-test, p<0.0001). As already pointed out by others [4], [24], this confirms that the STRF model poorly generalizes to another type of acoustic stimuli.

### Contribution of Bursts to STRF Differences

Differences between STRF_voc_ and STRF_dmr_ can result from nonlinearities in the responses which can have a different impact on the reverse-correlation performed on each stimulus set. We evaluated whether high frequency bursts (>200 Hz) of APs – which potentially constitute nonlinear responses – account for some of the differences between the two types of STRF. On average, evoked bursts represented 7.2% of the events at the presentation of vocalizations and 7.3% at the presentation of DMRs (ranging 0–38% in both cases). As bursts were made of at least two action potentials (AP), this means that, on average, for both set of stimuli, at least 15% of the total numbers of AP actually comes from bursts. There were significantly more bursts in layers III and V than in the other layers (10.4% and 8.95% respectively vs. 5.07 in layer I/II; 4% in layer IV and 5.1% in layer VI).

First, to evaluate whether bursts are responsible for the observed differences between STRF_voc_ and STRF_dmr_, we removed all bursts from the spike trains and recomputed the SI index between STRF_voc_ and STRF_dmr_. If bursts were responsible of the low SI values, their removal should improve the mean SI. The reverse was found: the SI between STRF_voc_ and STRF_dmr_ significantly decreased once bursts were removed (mean SI_without Burst_ = 0.10 vs. 0.15 with the bursts; paired t-test p<0.05) indicating that bursts contributed to increase, rather than to decrease, the similarity between STRF_voc_ and STRF_dmr_. Moreover, we evaluated whether bursts modify the response predictability by computing the CC_voc_ and CC_dmr_ on single AP responses. The removal of bursts did not change the CC values (CC_voc_ _without burst_ = 0.31 similar to CC_voc_ = 0.30, paired t-test, p>0.3, CC_dmr_ _without burst_ = 0.18 similar to CC_dmr_ = 0.17 paired t-test, p>0.3).

Despite these surprising results, we decided to go further by evaluating whether the low SI index between STRF_voc_ and STRF_dmr_ resulted from a difference in the number of bursts evoked by vocalizations and by DMRs. No correlation was observed between the difference in percentage of bursts (or the absolute value of this difference) and the SI value (r = 0.07, p = 0.67). There was also no correlation between the difference in number of spikes within bursts evoked by vocalizations and DMRs (or the absolute value of this difference) and the SI values (r = 0.02, p = 0.9). This lack of correlation suggests that the differences between STRF_voc_ and STRF_dmr_ cannot simply be explained by a difference in burst proportions evoked by vocalizations and by DMRs.

**Figure 8:**
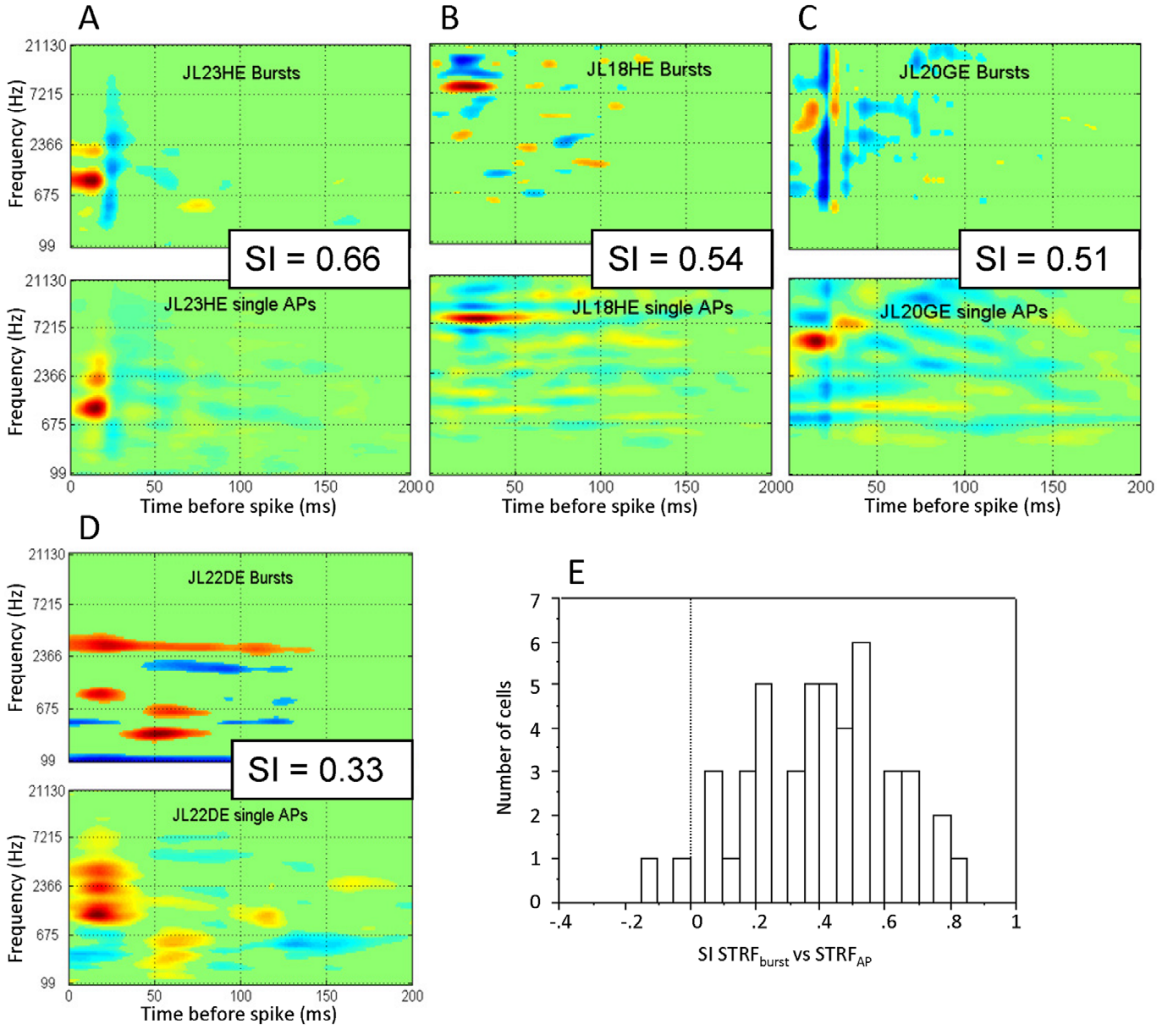
Comparison between STRFs obtained from single action potentials (STRF_AP_) and from bursts (STRF_Burst_). A., B. and C. show examples of cells exhibiting relatively similar STRF_AP_ and STRF_Burst_ (SI>0.5). In the three cases, the main excitatory zone is in the same frequency range for STRF_AP_ and STRF_Burst_; differences are mainly observed in small excitatory and inhibitory areas. Note that inhibition zones are more prominent in STRF_Burst_ than in STRF_AP_. D. Example of differences between STRF_AP_ and STRF_Burst_ (SI<0.5). The maximal excitatory zone is different for the Burst response than for the single APs response. E. Distribution of SI between STRF_AP_ and STRF_Bursts_ (mean SI = 0.39±0.22). The distribution shows a continuous range of SI values indicating that bursts of spikes can produce, on average, a STRF relatively similar to the STRF produced by single APs. STRF units and scale are the same as in Figure 2.

Nonetheless, the question remains whether evoked bursts constitute a nonlinearity detrimental to STRF calculation. For this purpose, we computed separately STRF from the single AP and from the burst component of each spike train. After applying the criterion to detect burst events (see Methods), we selected cells having more than one hundred evoked bursts for each stimulus set and, for each cell, we computed two STRFs: one with only single action potentials (STRF_AP_) and one with only bursts (STRF_Burst_). In total, 31/92 cells responding to vocalizations satisfied this criterion, and 23/92 cells did for the DMRs. Figure 8 shows examples of STRFs derived from bursts and single AP at presentation of vocalizations (8A and 8B) or of DMRs (8C and 8D). In three cases (Figure 8A–C), these examples show that the STRF_Burst_ is quite similar to the STRF_AP_, but with more prominent inhibitory zones in the STRF_Burst_. The STRFs presented in Figure 8D shows an example of dissimilarity between STRF_Burst_ and STRF_AP_. The distribution of SI values between STRF_AP_ and STRF_Burst_ did not differ from a normal distribution (Figure 8E) and its mean value was 0.39±0.22. There was no correlation (r = 0.097, p = 0.52) between the number of bursts and the similarity between the STRF_AP_ and STRF_Burst_, indicating that bursts generate STRFs which either match or differ from those generated by the single AP.

## Discussion

Testing guinea pig auditory cortex neurons with conspecific vocalizations and dynamic moving ripples (DMRs) allowed obtaining STRFs for a large proportion of cells (62% of the cells exhibiting reliable tuning curves showed significant STRF with vocalizations, 68% with DMRs). For 42 cells exhibiting significant STRFs with both vocalizations and DMRs, the BF, latency, bandwidth and global shape of the STRFs often differed between vocalizations and DMRs. The trial-to-trial temporal reliability of evoked responses was similar at vocalizations and DMRs but the predictability (indexed by CC_dmr_ and CC_voc_) was higher for vocalizations than for DMRs presentations. Cortical bursts, which potentially introduce nonlinearities in the evoked responses, cannot by themselves explain the differences between STRF_voc_ and STRF_dmr_.

### Differences between STRF_voc_ and STRF_dmr_

What can explain the large differences observed between STRF_voc_ and STRF_dmr_? When estimating a neuron’s STRF using reverse correlation, the stimulus parameter space should be adequately sampled and the recorded response reliable. If not, the STRF estimates will be noisy and unreliable. In the following, we aim at demonstrating that, even if some differences were present between our two stimulus sets, they cannot, *per se*, account for the differences between STRFs.

Potentially, some of the differences observed may originate from stimulus-dependent biases in the STRF estimation procedure. Indeed, although reverse correlation is, in principle, accounting for acoustical differences in the stimulus sets, one can still suspect that some biases are produced by the regularization procedure. More precisely, in each stimulus set, a regularization criterion specifies which spectral and temporal modulation frequencies are used to compute the stimuli autocorrelation. Since the regularization criterion is chosen based on the STRF’s prediction for each stimulus set, some degree of stimulus dependencies in STRF estimates can be expected. In our experiment, although the two sets of stimuli were matched in terms of average power and modulation spectrum, the following differences still existed: (i) conspecific vocalizations have frequent, rapid and large changes in instantaneous sound intensity across frequency bands which do not exist in DMRs and (ii) harmonics in vocalizations appear as linearly spaced peaks in the spectrum whereas ripples are sinusoidal spectral modulations on a logarithmic axis. However, stimulus set biases are probably not the main source of the observed differences between STRF_voc_ and STRF_dmr_ for the following two reasons.

Firstly, the differences between STRF_voc_ and STRF_dmr_ were much more pronounced for the real data than for the NHPP model. The mean value of the Similarity Index (SI) between STRFs was 0.62 in the NHPP model simulations, a much higher value than the average SI obtained in our cortical data (SI = 0.15). In fact, the linear spiking model was used to obtain an estimate of the STRF shape differences solely due to stimulus set bias. Since this model was closely matched to our real data (similar STRF parameters, spontaneous and evoked firing rate, spike timing reliability), an average SI of 0.62 constitutes the maximal value that can be obtained taking into account the bias in our stimuli sets. This strongly suggests that the differences between our stimuli sets cannot, in itself, account for the STRF differences.

Secondly, our results suggest that auditory cortex neurons did not show a linear spectro-temporal response. Indeed, if auditory cortical neurons were responding linearly, their STRF should be the best estimator of their response and the predictions should be as high as the inter-trial reliability. In that case, the CC_voc_ (and respectively the CC_dmr_) obtained should be equal to the CC_psth-psth_. In contrast, the majority of cells had significantly lower CC than CC_psth-psth_. We only observed predictions comparable to inter-trial reliability in a few cases: CC_voc_ ≈ CC_psth-psth_ for only 6/57 cells and CC_dmr_ ≈ CC_psth-psth_ for only 1/63 cells (see Figure 4A1–A2).

### Bursts do not Account for the STRFs Differences

The higher value of inter-trial reliability over the linear predictability strongly suggests a nonlinear behavior of auditory cortex neurons. We favor this hypothesis and consider that most of the differences observed in STRF_voc_ vs. STRF_dmr_ (as well as the relatively poor predictability of the STRF) originate from the response nonlinearities. To further investigate these nonlinearities, we examined the effects introduced by high frequency bursts. Putatively, neural bursts constitute a response nonlinearity since these events occur as a result of complex interactions of nonlinear processes on different time scales [42]–[44]. On average, spikes from bursts represented around 15% of the action potentials (AP) emitted by cortical cells. These bursts reflected sometimes similar, sometimes different components of the STRFs than the single AP. Our analysis shows that bursts cannot account for the differences between STRF_voc_ and STRF_dmr_. Indeed, when removing entirely the bursts, the predictions did not change, and the discrepancy between STRF_voc_ and STRF_dmr_ increased (the mean SI decreased from 0.15 to 0.10). This suggests that bursts occurred more frequently for acoustical events falling within the linear part of a neuron’s receptive field. This is in good agreement with the observations that auditory thalamus bursts occurred preferentially at the neurons’ BF [51]. It is also in good agreement with the results by Shih et al (2011): they showed that spikes contained in short interspike intervals (ISI) were more feature selective and conveyed more information than spikes contained in long ISI [53]. However, in that study, since a liberal criterion was set to detect potential bursts (ISI<15 ms), there is no guarantee that real “bursts” were detected and not simply accelerations of tonic firing rate.

Here, our criterion for isolating cortical bursts was based on parameters amply validated at the thalamic [46]–[51] and cortical level [33], [54]. Nonetheless, different types of bursts can be generated by different cortical cells (review in [55], [56]) and therefore the possibility of implementing cortical bursts in a model is not as straightforward as at the thalamic level [45]. In previous studies, differences in cortical cell types have been shown to produce different STRFs [57], [58]. At variance with these studies, no claim can be made here about differences according to cortical cell types, i.e., potential differences between regular spiking cells vs. thin spike cells. The very low proportion of thin spike cells obtained here (n = 4) compared with the large proportion of broad spike cells (n = 88) makes statistical analyses meaningless.

### Non Linearity and Context Dependence of Auditory Responses

Response nonlinearities leading to differences in STRFs have been investigated in an increasing number of studies [17]–[19], [22], [45], [59]. Initially, Theunissen and colleagues [4] proposed that although they observed differences in STRF obtained from bird songs and random tones, the nonlinear neural response was locally correctly approximated (i.e. within each stimulus set) by the estimated STRF. This hypothesis is corroborated by the good predictions obtained at different levels of the bird auditory system. An average CC of 0.51 and 0.45 was observed in the bird forebrain [4] and in sub-regions of the field L, the average CC ranged 0.37 to 0.63 [60]. A mean CC of 0.69 was even observed when testing field L neurons with artificial stimuli closely matching songbird acoustics [58]. When predictability is comparable to inter-trial reproducibility, one can use the STRF as a good linear estimate in each subset in order to better capture the full nonlinear neural response. Improvement of predictions was for instance demonstrated in the inferior colliculus by Lesica and Groethe [23] when using STRFs obtained at two different stimulus levels. Note that, at the level of the inferior colliculus, STRFs seem to provide a good predictability in all species (Zebra finch: mean CC = 0.4 in [21]; Gerbils: mean CC = 0.6 in [23]; Bat: median CC>0.3 in [61]).

Surprisingly, studies performed in the auditory cortex of mammals did not report such a high predictability. For example, using ensembles of natural stimuli, Machens et al. [22] showed that only 11% of the response power was predicted by the STRF linear model. Similarly, using artificial stimuli (dynamic random chords), Sahani and Linden [62] found that STRF models account for 18 to 40% of the stimulus related power in auditory cortex response; and more recently CC of 0.25 for speech-like stimuli and 0.13 for TORC stimuli were reported by David et al. [24]. Thus, with a mean CC of 0.29 for the STRF_voc_ and a mean value of 0.19 for the STRF_dmr_ our results are in the range of what was previously reported in mammalian auditory cortex.

This literature suggests that two factors contribute to the heterogeneity of the CC values. Firstly, the predictability seems to deteriorate when progressing from midbrain to cortex in mammals (see for example Lesica and Groethe [23] in the midbrain vs. Machens et al [22], David et al [24] in the cortex), maybe as a consequence of additional nonlinear processing occurring in the thalamo-cortical network. Secondly, the predictability seems to be higher for natural than for artificial stimuli. In fact, this is also true for our NHPP simulations: the CC values were slightly, but significantly higher, for vocalizations than for DMR (0.37 vs. 0.26 respectively). This suggests that, compared to artificial stimuli, communication sounds which contain amplitude modulations across frequency bands, produce a theoretical bias toward higher CC values. However, it remains, that in most cases, the predictions are far from been optimal at the cortical level. High values of temporal reliability were observed independently of the stimulus set, even though vocalizations had more frequent and rapid variations of sound intensity. Hence, the question remains of explaining what generates this deterministic stimulus-response function.

In fact, any arbitrary stimulus-response function can be approximated by Wiener kernel series, the STRF being the first order kernel. If higher order kernels are important in describing cortical neuron responses, then interactions between different spectro-temporal components matters. Many types of contextual effects have a strong impact on the neurons’ receptive field in auditory cortex: Additional peaks in the neuron’s STRF can be observed by reducing the stimulus spectral content [63] and multi-linear modeling has been shown to account for two-tones suppression and adaptation [18]. In fact, other types of contextual effects have a strong impact on the neurons’ receptive field. For instance, the attention for a target stimulus during a behavioral task can shift the STRF excitatory area [8], [9]; the shift from wakefulness to slow-wave sleep can shrink the receptive area of cortical and thalamic cells [33], [34], [64], [65]. These acoustical and state-dependent contextual effects point out that STRFs reflect a snapshot, not only of the neuron’s response, but also of the entire network converging on that particular neuron.

### Conclusion

Our results should be taken as additional evidence that cortical neurons, showing reliable tuning curves, process communication sounds in a way that cannot be predicted based on the responses obtained with artificial stimuli. This is probably not the result of a “learned” significance of these natural stimuli but most likely of the particular acoustic characteristics of these communication sounds and of the nonlinearity of the cortical responses. It was not the purpose of our study to dissect the mechanisms of cortical nonlinearities, but an original contribution of our work is to show that cortical high frequency bursts do not constitute nonlinearities detrimental to cortical STRF estimation. Further studies, combining the use of virtual vocalizations [66], electrophysiological recordings and modeling approaches, are required to understand to what extent the processing of communication sounds benefit from these cortical nonlinearities.

## Supporting Information

Figure S1 **Comparison between parameters obtained with pure tones and those derived either from STRF_dmr_ or from STRF_voc_.** A. These scattergrams show the values of the parameters derived from classical tuning curves (abscissa) against the values of the BF derived from STRF_dmr_ (ordinates). The values of the BF was similar (A1), the tuning bandwidth were slightly broader (A2) and the latency shorter (A3) when computed from STRF_dmr_ than with pure tones. B. These scattergrams show the values of the parameters derived from classical tuning curves (abscissa) against the values of the BF derived from STRF_voc_ (ordinates). In many cases, the values of the BF was lower (B1), the bandwidth was broader (B2) and the latency was shorter (B3) when computed from STRF_voc_ than with pure tones.
(TIF)

## References

[1] de Boer E, de Jongh HR (1978) On cochlear encoding: potentialities and limitations of the reverse-correlation technique. J Acoust Soc Am 63: 115–135. 632404 10.1121/1.381704

[2] Aertsen AM, Johannesma PI (1981) The spectro-temporal receptive field. A functional characteristic of auditory neurons. Biol Cybern 42: 133–143. 7326288 10.1007/BF00336731

[3] Eggermont JJ, Johannesma PM, Aertsen AM (1983) Reverse-correlation methods in auditory research. Q Rev Biophys 16: 341–414. 6366861 10.1017/s0033583500005126

[4] Theunissen FE, Sen K, Doupe AJ (2000) Spectral-temporal receptive fields of nonlinear auditory neurons obtained using natural sounds. J Neurosci 20: 2315–2331. 10704507 10.1523/JNEUROSCI.20-06-02315.2000 PMC6772498

[5] Klein DJ, Depireux DA, Simon JZ, Shamma SA (2000) Robust spectrotemporal reverse correlation for the auditory system: optimizing stimulus design. J Comput Neurosci 9: 85–111. 10946994 10.1023/a:1008990412183

[6] Kowalski N, Depireux DA, Shamma SA (1996) Analysis of dynamic spectra in ferret primary auditory cortex. I. Characteristics of single-unit responses to moving ripple spectra. J Neurophysiol 76: 3503–3523. 8930289 10.1152/jn.1996.76.5.3503

[7] Kowalski N, Depireux DA, Shamma SA (1996) Analysis of dynamic spectra in ferret primary auditory cortex. II. Prediction of unit responses to arbitrary dynamic spectra. J Neurophysiol 76: 3524–3534. 8930290 10.1152/jn.1996.76.5.3524

[8] Fritz J, Shamma S, Elhilali M, Klein D (2003) Rapid task-related plasticity of spectrotemporal receptive fields in primary auditory cortex. Nat Neurosci 6: 1216–1223. 14583754 10.1038/nn1141

[9] Fritz JB, Elhilali M, Shamma SA (2005) Differential dynamic plasticity of A1 receptive fields during multiple spectral tasks. J Neurosci 25: 7623–7635. 16107649 10.1523/JNEUROSCI.1318-05.2005 PMC6725393

[10] Escabi MA, Schreiner CE (2002) Nonlinear spectrotemporal sound analysis by neurons in the auditory midbrain. J Neurosci 22: 4114–4131. 12019330 10.1523/JNEUROSCI.22-10-04114.2002 PMC6757655

[11] Miller LM, Escabí MA, Read HL, Schreiner CE (2002) Spectrotemporal receptive fields in the lemniscal auditory thalamus and cortex. J Neurophysiol 87: 516–527. 11784767 10.1152/jn.00395.2001

[12] de Charms RC, Blake DT, Merzenich MM (1998) Optimizing sound features for cortical neurons. Science 280: 1439–1443. 9603734 10.1126/science.280.5368.1439

[13] Rutkowski RG, Shackleton TM, Schnupp JWH, Wallace MN, Palmer AR (2002) Spectrotemporal receptive field properties of single units in the primary, dorsocaudal and ventrorostral auditory cortex of the guinea pig. Audiol Neurootol 7: 214–227. 12097721 10.1159/000063738

[14] Linden JF, Liu RC, Sahani M, Schreiner CE, Merzenich MM (2003) Spectrotemporal structure of receptive fields in areas AI and AAF of mouse auditory cortex. J Neurophysiol 90: 2660–2675. 12815016 10.1152/jn.00751.2002

[15] Valentine PA, Eggermont JJ (2004) Stimulus dependence of spectro-temporal receptive fields in cat primary auditory cortex. Hear Res 196: 119–133. 15464309 10.1016/j.heares.2004.05.011

[16] Bitterman Y, Mukamel R, Malach R, Fried I, Nelken I (2008) Ultra-fine frequency tuning revealed in single neurons of human auditory cortex. Nature 451: 197–201. 18185589 10.1038/nature06476 PMC2676858

[17] Christianson GB, Sahani M, Linden JF (2008) The consequences of response nonlinearities for interpretation of spectrotemporal receptive fields. J Neurosci 28: 446–455. 18184787 10.1523/JNEUROSCI.1775-07.2007 PMC6670552

[18] Ahrens MB, Linden JF, Sahani M (2008) Nonlinearities and contextual influences in auditory cortical responses modeled with multilinear spectrotemporal methods. J Neurosci 28: 1929–1942. 18287509 10.1523/JNEUROSCI.3377-07.2008 PMC6671443

[19] Atencio CA, Sharpee TO, Schreiner CE (2008) Cooperative nonlinearities in auditory cortical neurons. Neuron 58: 956–966. 18579084 10.1016/j.neuron.2008.04.026 PMC2535914

[20] Woolley SMN, Fremouw TE, Hsu A, Theunissen FE (2005) Tuning for spectro-temporal modulations as a mechanism for auditory discrimination of natural sounds. Nat Neurosci 8: 1371–1379. 16136039 10.1038/nn1536

[21] Woolley SMN, Gill PR, Theunissen FE (2006) Stimulus-dependent auditory tuning results in synchronous population coding of vocalizations in the songbird midbrain. J Neurosci 26: 2499–2512. 16510728 10.1523/JNEUROSCI.3731-05.2006 PMC6793651

[22] Machens CK, Wehr MS, Zador AM (2004) Linearity of cortical receptive fields measured with natural sounds. J Neurosci 24: 1089–1100. 14762127 10.1523/JNEUROSCI.4445-03.2004 PMC6793584

[23] Lesica NA, Grothe B (2008) Dynamic spectrotemporal feature selectivity in the auditory midbrain. J Neurosci 28: 5412–5421. 18495875 10.1523/JNEUROSCI.0073-08.2008 PMC6670618

[24] David SV, Mesgarani N, Fritz JB, Shamma SA (2009) Rapid synaptic depression explains nonlinear modulation of spectro-temporal tuning in primary auditory cortex by natural stimuli. J Neurosci 29: 3374–3386. 19295144 10.1523/JNEUROSCI.5249-08.2009 PMC2774136

[25] Berryman JC (1976) Guinea-pig vocalizations: their structure, causation and function. Z Tierpsychol 41: 80–106. 961122 10.1111/j.1439-0310.1976.tb00471.x

[26] Berryman JC (1970) Guinea-pig voclizations. Guinea-Pig Newsletter 2: 9–18.

[27] King JA (1956) Social relations in the domestic guinea pig under seminatural conditions Ecology. 37: 221–228.

[28] Edeline JM, Pham P, Weinberger NM (1993) Rapid development of learning-induced receptive field plasticity in the auditory cortex. Behav Neurosci 107: 539–551. 8397859 10.1037//0735-7044.107.4.539

[29] Manunta Y, Edeline JM (1999) Effects of noradrenaline on frequency tuning of auditory cortex neurons during wakefulness and slow-wave sleep. Eur J Neurosci 11: 2134–2150. 10336682 10.1046/j.1460-9568.1999.00633.x

[30] Huetz C, Philibert B, Edeline JM (2009) A spike-timing code for discriminating conspecific vocalizations in the thalamocortical system of anesthetized and awake guinea pigs. J Neurosci 29: 334–350. 19144834 10.1523/JNEUROSCI.3269-08.2009 PMC6664951

[31] Wallace MN, Rutkowski RG, Palmer AR (2000) Identification and localisation of auditory areas in guinea pig cortex. Exp Brain Res 132: 445–456. 10912825 10.1007/s002210000362

[32] Wallace MN, Palmer AR (2008) Laminar differences in the response properties of cells in the primary auditory cortex. Exp Brain Res 184: 179–191. 17828392 10.1007/s00221-007-1092-z

[33] Edeline JM, Dutrieux G, Manunta Y, Hennevin E (2001) Diversity of receptive field changes in auditory cortex during natural sleep. Eur J Neurosci 14: 1865–1880. 11860482 10.1046/j.0953-816x.2001.01821.x

[34] Edeline JM, Manunta Y, Hennevin E (2000) Auditory thalamus neurons during sleep: changes in frequency selectivity, threshold, and receptive field size. J Neurophysiol 84: 934–952. 10938318 10.1152/jn.2000.84.2.934

[35] Manunta Y, Edeline JM (2004) Noradrenergic induction of selective plasticity in the frequency tuning of auditory cortex neurons. J Neurophysiol 92: 1445–1463. 15084638 10.1152/jn.00079.2004

[36] Slaney M (1998) Auditory Toolbox. Apple Tech. Report.

[37] Philibert B, Laudanski J, Edeline JM (2005) Auditory thalamus responses to guinea-pig vocalizations: a comparison between rat and guinea-pig. Hear Res 209: 97–103. 16139975 10.1016/j.heares.2005.07.004

[38] Singh NC, Theunissen FE (2003) Modulation spectra of natural sounds and ethological theories of auditory processing. J Acous Soc Am 114: 3394–3411. 10.1121/1.1624067 14714819

[39] Elhilali M, Fritz JB, Klein DJ, Simon JZ, Shamma SA (2004) Dynamics of precise spike timing in primary auditory cortex. J Neurosci 24: 1159–1172. 14762134 10.1523/JNEUROSCI.3825-03.2004 PMC6793586

[40] Huetz C, Negro CD, Lebas N, Tarroux P, Edeline JM (2006) Contribution of spike timing to the information transmitted by HVC neurons. Eur J Neurosci 24: 1091–1108. 16930435 10.1111/j.1460-9568.2006.04967.x

[41] Schreiber S, Fellous J, Whitmer D, Tiesinga P, Sejnowski T (2003) A new correlation-based measure of spike timing reliability Neurocomputing. 52–4: 925–931. 10.1016/S0925-2312(02)00838-X PMC2926980 20740049

[42] Rinzel J, Ermentrout B (1998) Methods in Neuronal Modeling: From Synapses to Networks. 251–291.

[43] Izhikevich E (2000) Neural excitability, spiking and bursting Int. J.Bif. Chaos 10: 1171–1266.

[44] Coombes S, Bressloff P (2005) BURSTING: The Genesis of Rhythm in the Nervous. (London) World Scientific Publishing Co. Pte. Ltd.

[45] Lesica NA, Stanley GB (2004) Encoding of natural scene movies by tonic and burst spikes in the lateral geniculate nucleus. J Neurosci 24: 10731–10740. 15564591 10.1523/JNEUROSCI.3059-04.2004 PMC6730113

[46] Guido W, Lu SM, Sherman SM (1992) Relative contributions of burst and tonic responses to the receptive field properties of lateral geniculate neurons in the cat. J Neurophysiol 68: 2199–2211. 1491266 10.1152/jn.1992.68.6.2199

[47] Guido W, Weyand T (1995) Burst responses in thalamic relay cells of the awake behaving cat. J Neurophysiol 74: 1782–1786. 8989413 10.1152/jn.1995.74.4.1782

[48] Ramcharan EJ, Gnadt JW, Sherman SM (2000) Burst and tonic firing in thalamic cells of unanesthetized, behaving monkeys. Vis Neurosci 17: 55–62. 10750826 10.1017/s0952523800171056

[49] Swadlow HA, Gusev AG (2001) The impact of ‘bursting’ thalamic impulses at a neocortical synapse. Nat Neurosci 4: 402–408. 11276231 10.1038/86054

[50] Swadlow HA, Gusev AG, Bezdudnaya T (2002) Activation of a cortical column by a thalamocortical impulse. J Neurosci 22: 7766–7773. 12196600 10.1523/JNEUROSCI.22-17-07766.2002 PMC6757983

[51] Massaux A, Dutrieux G, Cotillon-Williams N, Manunta Y, Edeline JM (2004) Auditory thalamus bursts in anesthetized and non-anesthetized states: contribution to functional properties. J Neurophysiol 91: 2117–2134. 14724263 10.1152/jn.00970.2003

[52] Gourévitch B, Eggermont JJ (2007) A nonparametric approach for detection of bursts in spike trains. J Neurosci Methods 160: 349–358. 17070926 10.1016/j.jneumeth.2006.09.024

[53] Shih YJ, Attencio CA, Schreiner CE (2011) Improved stimulus representation by short interspike intervals in primary auditory cortex. J Neurophysiol. 105: 1908–17. 10.1152/jn.01055.2010 PMC3075280 21307320

[54] Manunta Y, Edeline JM (2000) Noradrenaline does not change the mode of discharge of auditory cortex neurons. Neuroreport 11: 23–26. 10683823 10.1097/00001756-200001170-00005

[55] Markram H, Toledo-Rodriguez M, Wang Y, Gupta A, Silberberg G, et al (2004) Interneurons of the neocortical inhibitory system. Nat Rev Neurosci 5: 793–807. 15378039 10.1038/nrn1519

[56] PINGroup, Ascoli GA, et al (2008) Petilla terminology: nomenclature of features of GABAergic interneurons of the cerebral cortex. Nat Rev Neurosci 9: 557–568. 18568015 10.1038/nrn2402 PMC2868386

[57] Atencio CA, Schreiner CE (2008) Spectrotemporal processing differences between auditory cortical fast-spiking and regular-spiking neurons. J Neurosci 28: 3897–3910. 18400888 10.1523/JNEUROSCI.5366-07.2008 PMC2474630

[58] Nagel KI, Doupe AJ (2008) Organizing principles of spectro-temporal encoding in the avian primary auditory area field L. Neuron. 58: 938–955. 10.1016/j.neuron.2008.04.028 PMC2547416 18579083

[59] Escabí MA, Nassiri R, Miller LM, Schreiner CE, Read HL (2005) The contribution of spike threshold to acoustic feature selectivity, spike information content, and information throughput. J Neurosci 25: 9524–9534. 16221863 10.1523/JNEUROSCI.1804-05.2005 PMC6725702

[60] Sen K, Theunissen FE, Doupe AJ (2001) Feature analysis of natural sounds in the songbird auditory forebrain. J Neurophysiol 86: 1445–1458. 11535690 10.1152/jn.2001.86.3.1445

[61] Andoni S, Li N, Pollak GD (2007) Spectrotemporal receptive fields in the inferior colliculus revealing selectivity for spectral motion in conspecific vocalizations. J Neurosci 27: 4882–4893. 17475796 10.1523/JNEUROSCI.4342-06.2007 PMC6672083

[62] Sahani M, Linden JF (2003) How Linear are Auditory Cortical Responses? Advances in Neural Information Processing System 15: 109–116.

[63] Gourévitch B, Noreña A, Shaw G, Eggermont JJ (2009) Spectrotemporal receptive fields in anesthetized cat primary auditory cortex are context dependent. Cereb Cortex 19: 1448–1461. 18854580 10.1093/cercor/bhn184

[64] Issa EB, Wang X (2011) Altered neural responses to sounds in primate primary auditory cortex during slow-wave sleep. J Neurosci 31: 2965–2973. 21414918 10.1523/JNEUROSCI.4920-10.2011 PMC3758555

[65] Issa EB, Wang X (2008) Sensory responses during sleep in primate primary and secondary auditory cortex. J Neurosci 28: 14467–14480. 19118181 10.1523/JNEUROSCI.3086-08.2008 PMC3844765

[66] DiMattina C, Wang X (2006) Virtual vocalization stimuli for investigating neural representations of species-specific vocalizations. J Neurophysiol 95: 1244–62. 16207780 10.1152/jn.00818.2005

