# Supplementary figures and images for: Differences between Spectro-Temporal Receptive Fields Derived from Artificial and Natural Stimuli in the Auditory Cortex
Source: PLoS One. 2012 Nov 27;7(11):e50539. doi: 10.1371/journal.pone.0050539 (PMC3507792; doi:10.1371/journal.pone.0050539)

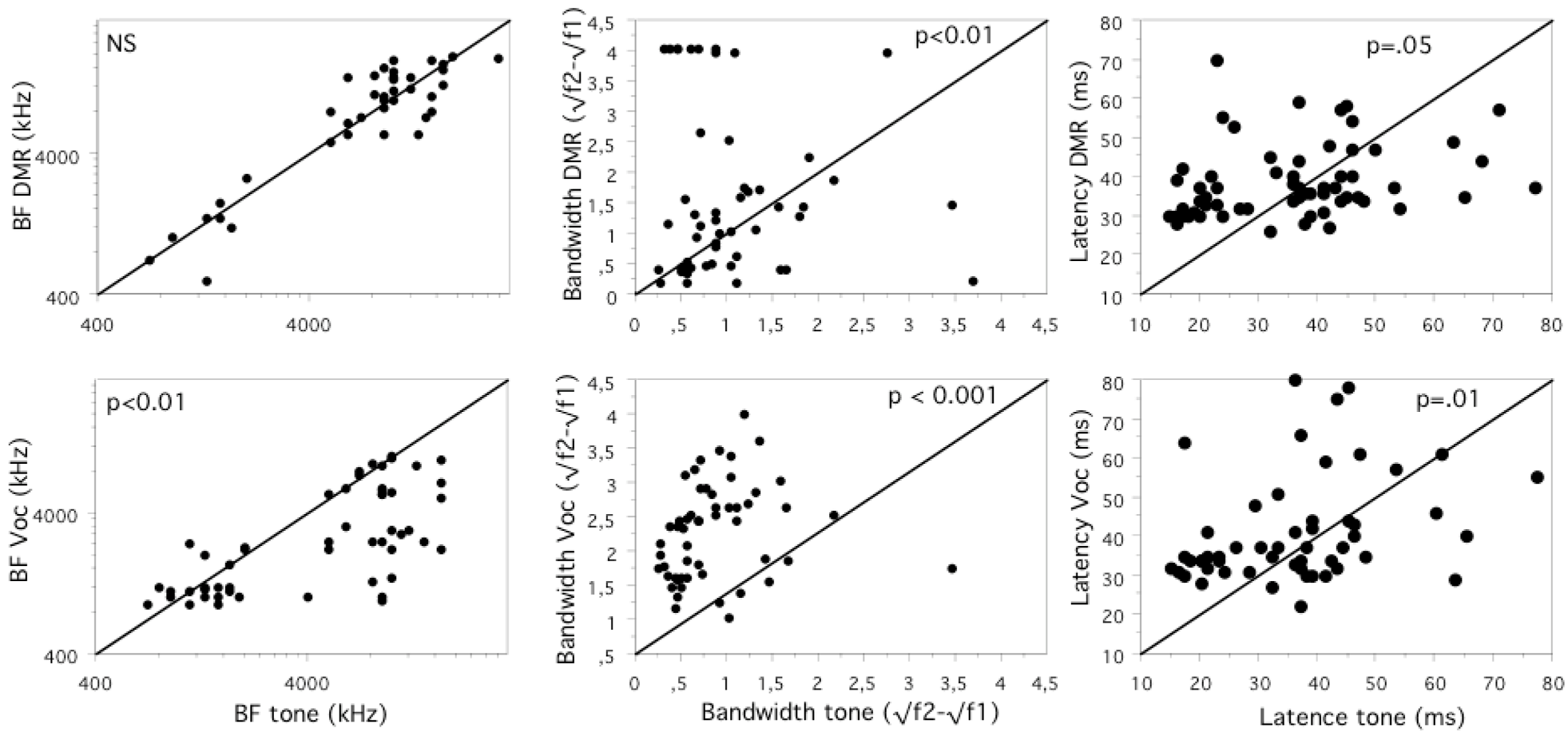

Supplement: Figure S1 — Comparison between parameters obtained with pure tones and those derived either from STRFdmr or from STRFvoc. A. These scattergrams show the values of the parameters derived from classical tuning curves (abscissa) against the values of the BF derived from STRFdmr (ordinates). The values of the BF was similar (A1), the tuning bandwidth were slightly broader (A2) and the latency shorter (A3) when computed from STRFdmr than with pure tones. B. These scattergrams show the values of the parameters derived from classical tuning curves (abscissa) against the values of the BF derived from STRFvoc (ordinates). In many cases, the values of the BF was lower (B1), the bandwidth was broader (B2) and the latency was shorter (B3) when computed from STRFvoc than with pure tones. (TIF) [file pone.0050539.s001.tif]
